# The social brain meets the reactive genome: neuroscience, epigenetics and the new social biology

**DOI:** 10.3389/fnhum.2014.00309

**Published:** 2014-05-21

**Authors:** Maurizio Meloni

**Affiliations:** School of Sociology and Social Policy, Institute for Science and Society, University of NottinghamNottingham, UK

**Keywords:** epigenetics, Meaney, methylation, neuroepigenetics, postgenomics, plasticity, social neuroscience

## Abstract

The rise of molecular epigenetics over the last few years promises to bring the discourse about the sociality and susceptibility to environmental influences of the brain to an entirely new level. Epigenetics deals with molecular mechanisms such as gene expression, which may embed in the organism “memories” of social experiences and environmental exposures. These changes in gene expression may be transmitted across generations without changes in the DNA sequence. Epigenetics is the most advanced example of the new postgenomic and context-dependent view of the gene that is making its way into contemporary biology. In my article I will use the current emergence of epigenetics and its link with neuroscience research as an example of the new, and in a way unprecedented, sociality of contemporary biology. After a review of the most important developments of epigenetic research, and some of its links with neuroscience, in the second part I reflect on the novel challenges that epigenetics presents for the social sciences for a re-conceptualization of the link between the biological and the social in a postgenomic age. Although epigenetics remains a contested, hyped, and often uncritical terrain, I claim that especially when conceptualized in broader non-genecentric frameworks, it has a genuine potential to reformulate the ossified biology/society debate.

## After gene-centrism: the new social biology

Profound conceptual novelties have interested the life-sciences in the last three decades. In several disciplines, from neuroscience to genetics, we have witnessed a growing (and parallel) crisis of models that tended to sever biological factors from social/environmental ones. This possibility of disentangling neatly what seemed to belong to the “biological” from the “environmental” and to attribute a sort of causal primacy to biological factors (equated with genetic) in opposition to social or cultural ones (thought of as being more superficial, or appearing later in the ontology of development) was part and parcel of very vocal research-programs in the 1990s. These programs were all more or less heirs of the gene-centrism of sociobiology: from evolutionary psychology, to a powerful nativism that was very influential in psychology and cognitive neuroscience with its obsessive emphasis on hardwiring culture or morality into the brain.

These programs have always received a barrage of criticisms from several intellectual traditions (Griffiths, 2009; Meloni, 2013a), particularly those with roots in ethology (Lehrman, 1953, 1970; Bateson, 1991; Bateson and Martin, 1999), and developmental biology (West and King, 1987; Griffiths and Gray, 1994; Gottlieb, 1997; Oyama, 2000a[1985],b; Oyama et al., 2001; Griffiths, 2002; Moore, 2003). However, never as in this last decade, we have had scientific evidence that the dichotomous view of biology vs. society and biology vs. culture is biologically fallacious (Meaney, 2001a).

Paradoxically, it was exactly the completion of the Human Genome Project that showed that the view of the gene as a discrete and autonomous agent powerfully leading traits and developmental processes is more of a fantasy than actually being founded on scientific evidence, as highlighted by the “missing heritability” case (Maher, 2008). The image of a distinct, particulate gene marked by “clearly defined boundaries” and performing just one job, i.e., coding for proteins, has been overturned in recent years (Griffiths and Stotz, 2013: 68; see also Barnes and Dupré, 2008; Keller, 2011). Although discussions are far from being settled, the work of the ENCODE consortium for instance has been crucial in showing the important regulatory functions of what, in a narrow “gene-centric view”, was supposed to be mere “junk DNA” (Encode, 2007, 2012; Pennisi, 2012). Not only does a very small percentage of the genome (less than 2%) act according to the classical definition of the gene as a protein-coding sequence, but most of the non-protein coding DNA in fact plays an important regulatory function. The genome is therefore today best described as a “vast reactive system” (Keller, 2011) embedded in a complex regulatory network with distributed specificity (Griffiths and Stotz, 2013). An important part of this regulatory network is involved in responding *to environmental signals*, which can cover a very broad range of phenomena, from the cellular environment around the DNA, to the entire organism and, in the case of human beings, their social and cultural dynamics.

To sum up a decade of empirical and conceptual novelties the conceptualization of the gene has become dynamic and “perspectival” (Moss, 2003), in what can be called the new “postgenomic view^1^”; it addresses genes as part of a broader regulative context, “embedded inside cells and their complex chemical environments” that are, in turn, embedded in organs, systems and societies (Lewkowicz, 2010). Genes are now seen as “catalysts” more than “codes” in development (Elman et al., 1996), “followers” rather than “leaders” in evolution (West-Eberhard, 2003; Robert, 2004). The more genetic research has gone forward, the more genomes are seen to “respond in a flexible manner to signals from a massive regulatory architecture that is, increasingly, the real focus of research in ‘genetics’” (Griffiths and Stotz, 2013: 2; see also Barnes and Dupré, 2008; Dupré, 2012).

As Michael Meaney (2001a: 52, 58) wrote more than a decade ago: “There are no genetic factors that can be studied independently of the environment, and there are no environmental factors that function independently of the genome… . At no point in life is the operation of the genome independent of the context in which it functions.” Moreover, “environmental events occurring at a later stage of development … can alter a developmental trajectory” making meaningless any linear regression studies of nature and nurture. Genes are always “genes in context”, “context-dependent catalysts of cellular changes, rather “controllers” of developmental progress and direction” (Nijhout, 1990: 444), susceptible to be reversed in their expression by individual’s experiences during development (Champagne and Mashoodh, 2009).

## Epigenetics

The recent surge of interest in molecular epigenetics is probably the most visible example of these conceptual changes in contemporary biology. After a delay of almost fifty years from its coining, epigenetics has become a “buzzword” in XXI century biology (Jablonka and Raz, 2009: 131): the vertical growth of publications in the field in the last decade certifies this epidemic of epigenetics (Haig, 2012; Jirtle, 2012). It is far from my intention to oversell the conceptual and evidential strength of a discipline still as embryonic, multiple, and contested as molecular epigenetics. Many things in epigenetics remain highly controversial and debated, and cautiousness in dealing with its relevance, especially for humans, remains a good scientific policy (Feil and Fraga, 2012). Moreover, the notion of epigenetics is elusive and plastic, meaning different things for different research contexts (Morange, 2002; Bird, 2007; Ptashne, 2007; Dupré, 2012; Griffiths and Stotz, 2013). Despite (or, more likely, just because of) this semantic ambiguity epigenetics prospers as a scientific and social phenomenon in need of careful reflective scrutiny (Meloni and Testa, in press).

Also, the genealogy of epigenetics in biological thought is complex, and its current molecular “crystallization” is the result of a series of important conceptual shifts (Jablonka and Lamb, 2002; Haig, 2012; Griffiths and Stotz, 2013). The notion was firstly coined by embryologist and developmental biologist C. H. Waddington (1905–1975) in the 1940s as a neologism from *epigenesis* to define, in a broader *non-molecular sense*, the “whole complex of developmental processes” that connects genotype and phenotype (reprinted in Waddington, 2012). For Waddington epigenetics was “the branch of biology which studies the causal interactions between genes and their products which bring the phenotype into being” (Waddington, 1968 see Jablonka and Lamb, 2002).

A parallel origin of the concept is having probably a stronger influence on the present understanding of epigenetics. This latter tradition originates with Nanney’s (1958) paper in *Epigenetic Control Systems*, and refers more specifically to the existence of a second non-genetic system, at the cellular level, that regulates gene expression (Nanney, 1958; see, Haig, 2004; Griffiths and Stotz, 2013).

It is this second narrower molecular meaning that is becoming increasingly influential in the contemporary literature (Griffiths and Stotz, 2013). This is why it is probably more correct to call contemporary epigenetics “molecular epigenetics” to differentiate it from the broader Waddingtonian sense and the developmentalist-embryological tradition in which the term was firstly conceived, although it is true that the two meanings are not in principle irreconcilable as they both emphasize the context (molecular or at the level of the organism) where genetic functioning takes place (Hallgrímsson and Hall, 2011).

In the present mainstream molecular sense, a rather standard and very often quoted definition of “epigenetics” is “the study of mitotically and/or meiotically heritable changes in gene function that *cannot be explained* by changes in DNA sequence” (my italics, Russo et al., 1996, quoted in Bird, 2007: 396; see also Feng and Fan, 2009). This definition in a negative form is pretty typical even in less technical books, where we find epigenetics called as the study of all the “long-term alterations of DNA that *don’t involve* changes in the DNA sequence itself” (Francis, 2011: X, my italics).

In a broader but still negative form, epigenetics can be defined as any “phenotypic variation *that is not attributable* to genetic variation” (Haig, 2012: 15, my italics). If we search for an operationally positive definition (more rare), we can call molecular epigenetics “the active perpetuation of local chromatin states” (Bird and Macleod, 2004 quoted in Richards, 2006: 395) or the self-perpetuation of gene expression “in the absence of the original signal that caused them” (Dulac, 2010: 729). The preferred recourse to a negative definition not only reflects the uncertainty surrounding the range and stability of epigenetic mutations, but more importantly it makes evident the difficulties of conceptualizing epigenetics in a way that might finally go beyond a gene-centric view of heredity and phenotypic development^2^.

DNA methylation, the addition of a methyl group to a DNA base that can silence gene expression, is the most well-known example of an epigenetic modification. Given its crucial function as regulator of gene expression, methylation has been defined as the “*prima donna*” of epigenetics (Santos, quoted in Sweatt, 2013). Other possible examples of epigenetic marks include histone modifications, alterations of chromatin structure, and gene regulation by non-coding RNA.

In evolutionary terms, epigenetic changes, far from being a biological anomaly, are fundamental for developmental plasticity, the “intermediate process” by which a “fixed genome” can respond in a dynamic way to the solicitations from a changing environment, and produce different phenotypes from a single genome (Meaney and Szyf, 2005; cfr. also Robert, 2004; Gluckman et al., 2009, 2011). Recent studies (Kucharski et al., 2008; Lyko et al., 2010) on the impact of DNA methylation on the development of different phenotypes between sterile worker and fertile queen honeybees (*Apis mellifera*) have shown the importance of epigenetic changes (via different nutrition in this case) on the mechanism underlying developmental plasticity.

Even more interestingly, these changes in gene expression (and the phenotypic alteration that results from it) have a twofold property whose importance in rethinking the nexus of biology and social factors cannot be underestimated: (1) some epigenetic modifications, like DNA methylation, can be maintained throughout life whereas others are susceptible to change even later in life being therefore reversible under certain circumstances; and (2) some epigenetic states, against established wisdom, appear to be transmissible inter-generationally.

Point 2 especially remains very controversial because received wisdom is that these epigenetic marks are reset at each generation and therefore incapable of offering the required stability to sustain transgenerational phenotypic changes. It is true that the issue of transgenerational epigenetic inheritance remains the source of more questions than answers so far (Daxinger and Whitelaw, 2010), but novel and interesting studies are challenging the established view of inheritance (Anway et al., 2005; Rassoulzadegan et al., 2006; Hitchins, 2007; Wagner et al., 2008; Franklin et al., 2010; Saavedra-Rodriguez and Feig, 2013) and pointing at the transgenerational effects on future generations (up to four) of environmental effects via epigenetic mechanisms in the two alternative forms of: (a) germline epigenetic inheritance (where the epigenetic mark is directly transmitted, see for instance Anway et al. (2005); and (b) experience-dependent non-germline epigenetic inheritance (where the epigenetic mark is recreated in each successive generation by the re-occurrence of the inducing behavior, or “niche recreation”: Champagne, 2008, 2013a, b; Champagne and Curley, 2008; Danchin et al., 2011; Gluckman et al., 2011).

Possible examples of these latter indirect or non germline epigenetic phenomena in humans include the often quoted research on transgenerational effects on chronic disease in individuals prenatally exposed to famine during the Dutch Hunger Winter in 1944–45 (Heijmans et al., 2008; Painter et al., 2008; Veenendaal et al., 2013). In the context of the growing interest in the developmental origins of chronic noncommunicable disease in humans (the so-called “developmental origins of health and disease”, DOHaD), epigenetic research is bringing to light how, during particularly plastic phases of development, environmental cues (for instance, in the above quoted example, levels of nutrition) set up stable epigenetic markers that shape (or “program”) the organism’s later susceptibility to disease (Gluckman et al., 2011).

In a broader evolutionary perspective, epigenetic marks, and DNA methylation in particular, are becoming recognized as “candidate mechanisms” (Kappeler and Meaney, 2010; see also Danchin et al., 2011) for *parental effects*, the phenomenon whereby exposures in one generation to certain environmental states (for instance in this case, famine) can affect the next generation’s phenotypes without affecting their genotypes (Badyaev and Uller, 2009; Danchin et al., 2011).

## Consequences for heredity

It appears evident even from this limited survey that the consequences of epigenetics for the notion of biological inheritance are profound. By challenging the idea that heredity is the mere transmission of nuclear DNA, epigenetics has opened the doors to a broader, extended view of heredity by which information is transferred from one generation to the next by many interacting inheritance systems (Jablonka and Lamb, 2005). Epigenetic variations act as a parallel inheritance system through which the organism can respond in a more flexible and rapid way to environmental cues and transmit to different cell lineages different “interpretations” of DNA information (*ibid*.).

It is no longer the mere DNA sequence that is transferred inter-generationally, but, expanding on the notions of “ontogenetic niche” coined in the 1980s (West and King, 1987), it is the whole “developmental niche” (Stotz, 2008), “the set of environmental and social legacies that make possible the regulated expression of the genome during the life cycle of the organism” (Griffiths and Stotz, 2013: 110). Taking seriously the idea of a developmental niche as the proper integrative framework for extended inheritance, as Griffiths and Stotz (2013) claim, means also understanding that environmental and social factors, not only merely “genetic” factors, “carry information in development” (*ibid.*: 179).

The environment is therefore now seen as directly inducing variations in evolution (Jablonka and Lamb, 2005), and its role as “initiator of evolutionary novelties” clearly recognized (see also Pigliucci, 2001; West-Eberhard, 2003; Pigliucci and Muller, 2010).

In sum, the narrow, gene-centric view of inheritance that was at the core of the Modern Synthesis in evolutionary thinking has been profoundly challenged and opened to a plurality of different non-genetic mechanisms (Bonduriansky, 2012; Bonduriansky and Day, 2009; Uller, 2013). By inviting one to think that “heredity involves more than genes”, and that “new inherited variations (…) arise as a direct, and sometimes directed, response to environmental challenge” epigenetic inheritance seems close to Lamarckian ideas of soft inheritance and inheritance of acquired features (Jablonka and Lamb, 1995: 1; see also Jablonka and Lamb, 2005; Gissis and Jablonka, 2011), although clearly the interpretation of epigenetics in such a broad and heterodox conceptual framework remains debated and controversial.

## Where epigenetics meets neuroscience

Some of the most influential studies that are behind the recent surge of interest in epigenetics originate from or directly cut-across neuroscience research. Epigenetic research offers a key missing link in the dynamic interplay between experience and the genome in sculpting neuronal circuits especially in critical period of plasticity (Fagiolini et al., 2009). It attempts to make visible the molecular pathway that explain how transient environmental factors can lead “long-lasting modifications of neural circuits and neuronal properties” (Guo et al., 2011).

The porousness of the brain to social signals has been at the core of social neuroscience since its beginning in the 1990s. I will focus here on three streams of research that have played a crucial role in taking this openness and plasticity of the social brain to a new level. Epigenetics in this sense can be seen as the climax of that very visible process of the “socialization” of biological and neurobiological concepts that we have witnessed in action in evolutionary thinking since at least the 1990s (Meloni, 2014).

### Molecular pathways of maternal care in the brain

In current epigenetic studies the story of how Michael Meaney, a neuroscientist and clinical psychologist at McGill, and Moshe Szyf, a molecular biologist and professor of pharmacology at the same McGill, met in a bar during a conference in Spain, has been told many times (Buchen, 2010; Hoag, 2011) to show the almost serendipitous encounter of a neurobiological perspective with a genetic one that is behind social epigenetic research. This interdisciplinary approach lies at the very core of Meaney’s group’s maternal care studies on the intergenerational transmission of stress and inadequate mothering in rodents (Meaney, 2001b), amongst the most known in all the epigenetic literature (along with Waterland and Jirtle’s studies on agouti mice: Waterland and Jirtle, 2003, 2004). Also the story of how this study was first rejected by *Science* and *Nature* is told to illustrate the impervious terrain that marked the beginning of epigenetic research.

Meaney et al.’s study, finally published as *Epigenetic programming by maternal behavior* in *Nature Neuroscience* (Weaver et al., 2004) has become a massively quoted article (with more than 2500 citations), almost an icon of the new linkage between behavioral exposures (in this case: maternal care and neonatal handling) and genetic expression/development in the brain.

The basic findings of the study are that increased licking and nursing activity by rat mothers altered the offspring DNA methylation patterns in the hippocampus, thus affecting “the development of hypothalamic-pituitary-adrenal responses to stress through tissue-specific effects on gene expression” (Weaver et al., 2004: 847). Even more interestingly, cross-fostering pups of non-caring mothers to affective ones, the DNA methylation phenotype reflected that of the foster mother and was maintained stably into adulthood thus shaping life-long behavioral trajectories.

This direct linkage between maternal care and neurological development (via DNA methylation) was conceptualized in terms of environmental (or epigenetic) programming that is a stable non-sequence based modification (Francis et al., 1999) of gene expression that proceeds without germline transmission. Another take-home message of Meaney’s group study is the emphasis on a critical period, the first week of life, for the effects of early experience on methylation patterns in the hippocampus. Epigenetic modifications are stably encoded during early life experiences becoming therefore the critical factor in “mediating the relationship between these experiences and long-term outcomes” (Fagiolini et al., 2009). The sustained effects of these cellular modifications “appear to form the basis for the developmental origins of vulnerability to chronic disease” (Meaney et al., 2007).

### Stigmas of trauma in the brain

But what about epigenetic research involving more specifically humans? In 2009, another study appeared with a significant impact on the field of social epigenetic. The research, originating again from Meaney’s lab, focused on the level of DNA methylation in postmortem hippocampal tissue from two groups of suicide victims (using samples from the Quebec Suicide Brain Bank), one of which with a history of abuse (McGowan et al., 2009).

The study found higher levels of DNA methylation of the regulatory region of the glucocorticoid receptor (resulting in decreased levels of glucocorticoid receptor mRNA) in the abused group compared to the nonabused and the control group. Early life adversities therefore (childhood abuse), not suicide *per se*, are the key factors to explain the alteration of DNA methylation in crucial genomic regions (neuron-specific glucocorticoid receptor gene, NR3C1) in the brain.

This work, which translates Meaney’s research into human studies for the first time, is consistent with the findings of the studies on rodents and has been welcomed as biological evidence of how traumatic life experiences become embedded in the “memory” of the organism, getting “under the skin” (Hyman, 2009).

The findings of this research, along with others of McGowan et al. (2008), are consistent with the non-human animal studies of Meaney’s group about the emphasis on early life events as a critical period for the establishment of stable DNA methylation patterns, and therefore different pathways of neural development. As the study claims: “early life events can alter the epigenetic state of relevant genomic regions, the expression of which may contribute to individual differences in the risk for psychopathology” (McGowan et al., 2009: 346).

Like in Meaney’s group previous studies, the emphasis is on the effects of disruption of parental care on methylation levels in critical areas of the brain implicated in the regulation of responses to stress and anxiety disorders. More importantly, the study aims to open up important connections between variations in DNA methylation in the hippocampus and the emergence of psychiatric disorders, a topic that is becoming increasingly relevant in epigenetic research (see for instance Tsankova et al., 2007; Nestler, 2009), as it can be seen from the third and final cluster of what can be named “epigenetic neuroscience” research.

### Neuroepigenetics: mechanisms of plasticity for the adult brain

A final and parallel development at the crossroads of epigenetics and neuroscience comes from the newborn sub-field of (cognitive) *neuroepigenetics* (Day and Sweatt, 2011; Sweatt, 2013) that focuses on how epigenetic mechanisms impact the adult brain and the central nervous system.

Neuroepigenetics aims to investigate changes in epigenetic marks that accompany neuronal plasticity and the processes of learning and memory formation/maintenance in the brain (see also Levenson and Sweatt, 2005; Borrelli et al., 2008). In a sense, epigenetic marking itself can be seen as a “persistent form of cellular memory” by which memories of past environmental events are fixed on the genome. This would explain, it has been claimed, the fact that the nervous system has co-opted this mechanism “to subserve induction of synaptic plasticity, formation of memory and cognition in general” (Levenson and Sweatt, 2006). Another task of neuroepigenetics is the understanding how epigenetic mechanisms may vary depending on the different neural circuits and behavioral tasks involved (Day and Sweatt, 2011). The main difference compared to the other studies highlighted in this section is the emphasis on the adult brain. Here, given the non-divisibility of adult neuron cells, epigenetic tags although long-lasting are non-heritable, thus setting “the roles of epigenetic mechanisms in adult neurons apart from their roles in developmental biology” (Sweatt, 2013: 627). The term neuroepigenetics is what distinguishes therefore this specific aspect of epigenetic research from other areas of developmental biology (Day and Sweatt, 2011). A new wave of publications on the epigenetics of the adult brain illustrates well the high expectations surrounding epigenetic knowledge to explain the molecular mechanisms of plasticity. In a recent article, for instance, Woldemichael et al. (2014) look at the way epigenetic processes may subserve brain plasticity in relation to, amongst other things, drug addiction and cognitive dysfunctions (age-associated cognitive decline, Alzheimer’s disease, etc.). Moreover, they do so always with an eye to the potential of epigenetic therapies to reverse neurodegenerative disorders (see also Gapp et al., 2014). Other recent publications in the field examine the epigenetics of stress vulnerability and resilience (see also Stankiewicz et al., 2013; Zannas and West, 2014), neuropsychiatric disorders (Hsieh and Heisch, 2010), major psychosis (Labrie et al., 2012), autism spectrum disorders (Ptak and Petronis, 2010), mood disorders (Fass et al., 2014); again, with an eye to the development of novel therapeutics.

Although many of these publications reflect very early attempts to use epigenetic knowledge to explain the molecular mechanisms of brain plasticity, and although in much of this literature the supposed distinctiveness of epigenetic changes in the brain rather than in other organs is never really problematized, it is still helpful to survey this emerging literature as an illustration of the current process of rewriting, in epigenetic terms, of many themes from the last decade of research about the social brain, particularly its plasticity and permeability to environmental signals. Epigenetics in this sense can be seen as *the last frontier in the construction of the narrative about the sociality of the brain*, the discovery of a possible crucial mechanism mediating between environmental exposures, gene expression and neuronal development, that is likely to validate and give further strength, at the molecular level, to many of the intuitions that have been at the core of social neuroscience research since the 1990s.

## Implications for social theory

In the last two decades of research in cognitive science, mind and cognition have been understood increasingly as an extended, enacted and embodied phenomena (Clark and Chalmers, 1998; Thompson, 2007; Clark, 2008; Noë, 2009; Menary, 2010). Neuroscience has joined this trend: the brain has ceased to be represented as an isolated organ and instead become a multiply connected device profoundly shaped by environmental influences. One of the membranes demarcating the biological from the social, the skull (Hurley in Noë, 2009), has been made increasingly permeable to a two-way interaction.

The brain is increasingly thought of as a tool specifically designed to create social relationships, to reach out for human relationships and company, literally made sick by loneliness and social isolation (Cacioppo and Patrick, 2008; Hawkley and Cacioppo, 2010). The emergence of this novel language certifies to the success of a discipline like social neuroscience (Matusall et al., 2011), with its landscape populated by empathic brains and moral molecules, mirror neurons and plastic synapses.

However, in the context of this trend toward an increasing openness of the biological to social signals, the rise of molecular epigenetics *promises to bring this discourse to an entirely new and more powerful level*. Undoubtedly, this promissory vocabulary, which has always been part of the rhetoric of the life-sciences (as highlighted by a consistent body of scholarship in Science and Technology Studies), has not to be taken at face value. The “economy of hope” that surrounds epigenetics as a possible relaunch of the genomics discourse is in particular something that deserves critical scrutiny (Meloni and Testa, in press). However, the appreciation of this more critical moment, cannot become a reason to deny the potential contained in the epigenetic discourse, especially when conceptualized in more sophisticated non gene-centric frameworks (Griffiths and Stotz, 2013).

When compared with recent arguments about the sociality of the brain, epigenetics seems to play a twofold function. Epigenetics not only supplements social neuroscience by highlighting the molecular mechanisms that orchestrate brain plasticity and memory formation, but also seeks to blur any residual distinction between biology and social/ecological contexts. If the first model of the cognitive brain was that of a computing machine, entirely severed from environmental influences, and the brain of social neuroscience still oscillated between plastic change and hardwiring metaphors, with the rise of what can be named the “epigenetic brain” or neuroepigenetics research the reciprocal penetration of the social and the biological reaches a point where trying to establish any residual distinction seems increasingly a meaningless effort.

Particularly when conceptualized within theoretical frameworks like Developmental Systems Theory (Oyama, 2000a[1985], b; Oyama et al., 2001) and other postgenomics approaches, epigenetic research illustrates exemplarily how we are moving toward a post-dichotomous view of biosocial processes that research in social neuroscience was only partially able to anticipate. With the rise of molecular epigenetics, the biological is opened to environmental influences, to social factors, and to the marks of personal experience like never before. The sovereign role of the gene has been decentralized (Van Speybroeck, 2002) and the genome made a “reactive genome” (a term first coined by Gilbert, 2003, and expanded on more recently by Keller, 2011; Griffiths and Stotz, 2013).

At the same time the notion of vitality has been expanded to a new range of actors and “democratized” (Landecker and Panofsky, 2013). In epigenetic research, the “social” seems to assume a causative role in human biology to a degree unseen before (Landecker and Panofsky, 2013). The same emergence of a new terminology of “social and environmental programming” reflects this unprecedented prominence of the social level. Such a discourse was quite unimaginable under the Weismannian’s conception of an impenetrable barrier between soma and germ-line, as well in what can be seen as the molecular translation of Weismann’s argument (Griesemer, 2002) in the so-called Central Dogma of Molecular biology (Crick, 1958) which stated the strict one-side flow of information from DNA to RNA. In reversing the informational asymmetry between genotype and phenotype, in stressing the relevance of context (interpretation) upon the level of DNA information (Jablonka and Lamb, 2005; Jablonka and Raz, 2009) and finally in giving a life-span to genetic process, making them radically dependent on temporal factors (Landecker and Panofsky, 2013), epigenetics displays unique features that promise to radically change the language of biology and, as a consequence, the system of rules that have so far regulated the biology/society boundary.

On one level, this unprecedented porousness of the biological to the social comes as a good news for social scientists with an interest in notions of embodiment and in exploring the pathways through which the social shapes and is literally inscribed into the body. The investigation of the ways in which social structures and socio-economic differences literally get *under the skin* (*and in the brain*), affecting the deep recesses of human physiology, has always been an important concern of sociological theory, from the French doctor and economist René Villermé and Friedrich Engels in the 1800s (see Krieger and Davey Smith, 2004), to social epidemiologists (Krieger, 2001, 2004, 2011; Shaw et al., 2003; Krieger and Davey Smith, 2004) and neuroscientists (Lupien et al., 2000; Noble et al., 2005, 2007, 2012; Farah et al., 2006; Kishiyama et al., 2009; Hackman et al., 2010; Rao et al., 2010) in the early twenty-first.

However, given the epistemological and political implications of gene-centrism and the mainstream view of biology as an unchangeable form of secular destiny in the twentieth-century, these more plastic biosocial approaches have remained so far exceptions (Boas, 1910 research on the changing bodily form of immigrants and their descendants in the USA, being one of these exceptions). Under these unfavourable epistemic circumstances, the possibility of sophisticated and enriching biosocial explorations has been profoundly limited and mostly faced with skepticism by social theorists. To import the biological into the social, across the twentieth century, meant almost exclusively refer to unacceptable class, race or gender biased explanations. Facing this view of biology, disembodied social constructionist explanations that rejected biology entirely seemed (almost) the only way out for social scientists.

However, in the present scenario marked by the rise of epigenetics and the new social biology, this marginalization no longer seems compulsory for social scientists. Undoubtedly, epigenetics is likely to revitalize a social science approach interested in how “phenomena of the outside (….) undergo transformations and are incorporated to re-appear or be reproduced on the *inside”* (Beck and Niewöhner, 2006: 224; Niewöhner, 2011; Guthman and Mansfield, 2012). It may supplement various findings from medicine, neuroscience, and various animal studies on the way in which social phenomena (social position, socio-economic status (SES), social isolation, rank, stress, etc.) are translated into the body and affect human health. On these novel bases, a fresh dialog between social and biological disciplines in which epigenetics can penetrate the “sometimes obdurate wall between the life and social sciences” (Landecker and Panofsky, 2013: 2) seems more realistic than in the past (Rose, 2013; Meloni, 2013b, 2014).

On the other level, however, a recognition of the great potential of epigenetic research to reframe and go beyond the sterile nature/nurture opposition, is no reason to deny the ambiguities and contradictory claims aligning in the field, and the difficult methodological and epistemic questions still awaiting to be answered before any major biosocial synthesis may be proposed.

Even leaving aside hypes and controversies surrounding epigenetics, social scientists and theorists need to be aware that an entire new array of problems is emerging in the postgenomic scenario. This new complex of social problems does not derive from the dichotomous separation of biological and social causes in which the biological is supposed to have a causal primacy (as in the hostile post 1970 debates on sociobiology, genetic reductionism, or evolutionary psychology). Rather they arise for the exact opposite reason, that is, *because of the inextricable mixture* of social and biological factors typical of the epigenetics and postgenomic conceptual landscape.

There is a specific and in a way unprecedented profile of problems in the postgenomic age (Meloni, 2013b, 2014; Meloni and Testa, in press) that without any ambition to be conclusive I will try to sketch below. Rather than as consolidated analyses of what is likely to happen in the epigenetic era, though, these different clusters of problems can be read as preliminary questions for a possible agenda of the social studies of the life-sciences in the future years.

### Postgenomic epistemology: molecularizing nurture?

Epigenetic research undermines the nature/nurture opposition on both sides of the dichotomy. To the extent that genes are now “defined by their broader context”, our understanding of nature becomes less essentialist and “more epigenetic” (Griffiths and Stotz, 2013: 228), that is, always entangled with social and environmental factors. However the epistemic conditions for environmental, social or experiential factors to become readable in the epigenetic paradigm is their translation into signals at the molecular level (Landecker, 2011). This trend finds confirmation in the fact that different social categories (from race to class), and environmental factors (from maternal care, to food and toxins) are being increasingly conceptualized today in molecular terms (Landecker, 2011; Niewöhner, 2011).

Only to the extent that our understanding of nurture becomes more “mechanistic” (Griffiths and Stotz, 2013: 5) can we therefore find a solution to the nature/nurture conundrum in the postgenomic era. It is important to notice here that mechanisms are understood by Griffiths, Stotz and other philosophers of biology not as a vulgar reductionist concept but as a more sophisticated, multilevel, and emergentist notion which includes looking “upward to higher levels” (Bechtel, 2008: 21) as well as making room for the active, autonomous role of human agency.

This new version of mechanism, as Griffiths and Stotz again claim, is producing an unexpected rapprochement with themes from the holistic tradition, or as they prefer “integrationist” (*ibid*.: 103).

Nonetheless, although social scientists will recognize in this anti-reductionist rethinking of the notion of mechanism an appealing theoretical move, two sources of skepticism remain to be addressed: (1) that in spite of the many sophistications of philosophers of science and biology, the bulk of epigenetic research will much more naively try to do business as usual, inscribing the effects of complex social phenomena at the digitalized level of methylation marks (Meloni and Testa, in press), with serious risk of over-simplification as well as attributing causal relevance to random biological processes; and (2) that mainstream social theory will remain not convinced by any idea of the tractability of social and cultural phenomena, given the legacy of traditions (from Weberian neo-Kantism to Durkheim, from Western Marxism to Boasian anthropology: Benton, 1991; Meloni, 2011, 2014) that made anti-naturalism and the incommensurable nature of social and cultural processes the hallmark of social research.

Given these opposite limitations, complex biosocial and biocultural approaches are likely to remain a minority strategy, caught between persisting reductionist tendencies in bioscience and the continuing legacy of bio-phobia in social theory.

### Postgenomic biopolitics: “upgrade yourself” or born damaged for ever?

The epigenome is caught in a curious dialectic of stability and modifiability (Meloni and Testa, in press). Whereas genetic sequences are fixed and unchangeable, epigenetic marks are at the same time “long lasting” but “potentially reversible” (Weaver et al., 2005; McGowan and Szyf, 2010). In its social dimension, the plasticity of the epigenome, just like the plastic brain which Catherine Malabou (2008) has written about, can be understood in two alternative ways: (i) passively, as a capacity to receive form: the epigenome, in contrast to genes, is vulnerable to environmental insults; (ii) actively, as a capacity to give form: the epigenome can change and upgrade, through diet, exercise, therapeutic and social manipulations.

In the wider society, this dialectic within the language of epigenetics is likely to become even more amplified as an oscillation between determinism and hopes of individual/social amelioration: (i) determinism, because of the concerns that social and environmental insults can leave indelible scars on the body and brain (“Babies born into poverty are damaged forever before birth” titled the UK newspaper *The Scotsman* (Mclaughlin, 2012), to comment on a research on levels of methylation amongst different social groups in Glasgow, of which more below); (ii) amelioration, because the upgradable epigenome may become the basis for a new motivation to intervene, control and improve it through pharmacological agents or social interventions.

On the first dimension, political theorists and bioethicists have already started to reflect upon the “collective responsibility” to protect the vulnerable epigenome (Dupras et al., 2012; Hedlund, 2012) while legal theorists are speculating on the “number of novel challenges and issues” that epigenetic transgenerational effects may represent as a new possible “source of litigation and liability” (Rothstein et al., 2009: 37). The transmissibility via the epigenome of the insults of the past into the bodies of present or future generations raises therefore novel issues of *intergenerational equity*. This possible moralization of behaviors around the vulnerable epigenome is having a particularly visible example on the overwhelmingly centrality of the maternal body as a target of responsibility for harmful epigenetic consequences on the child’s health (Richardson, in press).

The second pole of this dialectic of plasticity, is instead represented by the many injunctions (it is enough to surf the web for some minutes to find many examples) to “upgrade”, “improve”, “train” or “change your epigenome”. The possibility of influencing the epigenome through diet, lifestyle, physical activity, stress, tobacco, alcohol, and pharmacological intervention becomes the likely basis for new forms of “therapeutic manipulations” (McGowan and Szyf, 2010). In David Shenk’s recent *The Genius in All of Us* one can see iconically the mobilization of epigenetics, celebrated as a “new paradigm” and “the most important discovery in the science of heredity since the gene” (Shenk, 2010: 129), at the service of a view of unlimited plasticity and constant struggle to enhance our capacity to reach talent and brilliance (see for a comment, Papadopoulos, 2011).

Which of the two poles of this dialectic of plasticity is going to prevail in the representation of epigenetics in the wider society, and in the shaping of epigenetic science itself, remains an open question. Science and society are constantly co-produced: this two-way interaction seems particularly visible in epigenetic research, thus representing a great opportunity to make of this newly emerging discipline a theoretical spyglass to observe the vivid emergence of the tensions and complexities of the postgenomic age.

### Postgenomic social policies?

The increasing emphasis on the biological embedding of life’s adversities at the genomic level is bringing to public attention what has been called a new “biology of social adversity” (Boyce et al., 2012). Epigenetic mechanisms are a major part of this novel approach. Epigenetics has already been used in the service of explaining the persistent nature, within specific groups, of “connections that have previously been hard to explain” (Landecker, 2011), particularly the perpetuation of health disparities between the rich and the poor, between and within countries (Vineis et al., 2013). An important trend is the use of epigenetic and developmental findings in the so-called early-intervention programmes (Shonkoff et al., 2009).

Over the last few years, a new array of studies has started to look at the way in which social influences can become embodied via epigenetic mechanisms and have lifelong and even inter-generational effects (Miller et al., 2009; Wells, 2010; Borghol et al., 2012). Kuzawa and Sweet (2009) study on racial disparities in cardiovascular health in the USA is a major example of the reconfiguration of the relationship between biological and social factors brought about by epigenetics. This work has focused on epigenetic and other developmental mechanisms as the missing link between early life environmental factors (e.g., maternal stress during pregnancy) and adult race-based health disparities in “hypertension, diabetes, stroke, and coronary heart disease”. It is an important attempt to rethink race along a different, somatic *and* socio-cultural together, line of thought.

In the UK, the study of McGuinness et al. (2012) on the correlation between SES and epigenetic status (variations in the level of methylation) between socio-economically deprived and more affluent groups in Glasgow (but also between manual and non-manual workers) points more empirically to an association between social neglect, poverty, and “aberrant” levels of methylation. “Global DNA hypomethylation” the study claims “was associated with the most deprived group of participants, when compared with the least deprived”. Epigenetic markers are used in this and other studies as a “bio-dosimeter” (*ibid*., 157) to measure the impact of social adversity on lifestyle and disease susceptibility (see also: Landecker and Panofsky, 2013).

Looking at the past two decades of attempts to use genetics and neuroscience in the public arena as the ultimate bastion of *evidence* for social deprivations and inequalities, it is possible that epigenetic findings will become increasingly relevant in social policy strategies. How these findings will help convince policy-makers of the “non-ethereal” nature of environmental influences in order to make “more effective arguments” about the biological impact of social forces (Miller, 2010), and influence specific political agendas (as seen in the notion of neuropolicy, see Racine et al., 2005) is difficult to foresee at this stage. It is clear however that the seductive appeal of neurobiological explanations (Wastell and White, 2012) is likely to be amplified further when combined with the seductive appeal of epigenetics, where social differences and environmental insults are expected now to be seen literally “imprinted on DNA”.

It is important however to remember the huge gap existing between public sensationalism, especially in its public health implication, and the cautious takes of the experts (Feil and Fraga, 2012; Meloni and Testa, in press). Even more ambiguously, the emergence of a possible discourse that identifies, at the local level, subgroups with abnormal epigenetic marks (reflecting the perpetuation of historically disadvantageous conditions) may create a whole new set of social and public policy questions. The legacy of soft or Lamarckian inheritance in social policy discourses has not always been particularly progressive (Bowler, 1984), and its possible returning appeal today should become a matter of reflection for social scientists (Meloni and Testa, in press). Moreover, there is increasing concern among social scientists that constructs rather widespread in epigenetics and DOHaD literature, from “maternal capital” (Wells, 2010) to the growing emphasis on maternal behaviors and the maternal body as the “vector” through which epigenetic patterns are established in early life (as highlighted by Richardson, in press), could have problematic effects on public health strategies and moral reasoning about families, parenting, and women in particular.

## Conclusion

In spite of my emphasis on some ambiguities of epigenetic research, the most important lesson for social scientists and theorists at this stage is probably that the future and therefore the social meaning of postgenomics and epigenetics *is not already written*. As Michel Morange (2006: 356) has claimed some years ago: “the very fashionable post-genomic programs can have very different stakes, some reductionist and other holistic, depending upon who is supporting them. The current state of biological research is very contrasted, because biology is hesitating at a crossroads between reductionism and holism”. It is therefore too early to say if molecular epigenetics will become mired in another form of reductionism (Lock, 2005) or will join new exciting theoretical collaborations capable to “transcend the divide between ‘nature’ and ‘nurture’ intellectually and methodologically” (Singh, 2012). Epigenetics is not set in stone, but an open field where theoretical debates and critiques are vital (Landecker and Panofsky, 2013). Given the multiple and plastic nature of its same concept, at the crossroads of different traditions and research-styles, epigenetics will likely be a terrain for conceptual battle between different stakeholders and intellectual agendas. This is probably one further reason for social scientists to be part of this debate from its very beginning.

## Conflict of interest statement

The author declares that the research was conducted in the absence of any commercial or financial relationships that could be construed as a potential conflict of interest.

## Footnotes

^1^Here postgenomics has to be understood in a twofold meaning: *chronologically* it refers to what has happened after the deciphering of the Human Genome in 2003; *epistemologically* it illustrates the emergence of a number of gaps in knowledge and unforeseen complexities surrounding the gene that has led to the current contextual conceptualization of the genome as affected by environmental signals and part of a broader regulative architecture (Dupré, 2012; Griffiths and Stotz, 2013). It is particularly this latter meaning that is central here.

^2^I thank one of the two anonymous reviewers for bringing this to my attention.
